# Surface Engineering & Coating Technologies for Corrosion and Tribocorrosion Resistance

**DOI:** 10.3390/ma16134863

**Published:** 2023-07-06

**Authors:** Yong Sun

**Affiliations:** School of Engineering and Sustainable Development, Faculty of Computing, Engineering and Media, De Montfort University, Leicester LE1 9BH, UK; ysun01@dmu.ac.uk

Corrosion of materials not only accounts for about 3 to 4% of economic losses in GDP in an industrial nation, but it also contributes significantly to greenhouse emissions and climate change because material production is one of the largest greenhouse emitters. According to a recent research study [1], steel production will account for 27.5% of carbon emissions worldwide by 2030 and corroded steel replacement will account for up to 9% of these emissions. Therefore, from economic and environmental points of view, it is highly necessary and important to enhance the corrosion and tribocorrosion resistance of materials. Since corrosion and tribocorrosion are surface- and subsurface-related material degradation phenomena, surface engineering and coating technologies are the most effective to tackle the corrosion and tribocorrosion problems of a wide range of engineering materials.

This Special Issue aimed to bring together the latest development in this technologically, economically and environmentally important area, encompassing coating development, corrosion and tribocorrosion characterization and industrial applications. It provides a forum for researchers to share their original work or insight reviews on “Surface engineering and coating technologies for corrosion and tribocorrosion resistance”.

This Special Issue contains 12 original research and review papers related to this topic, which have been contributed by researchers from around the globe. A brief description of these published works, which I am honored to edit as a Guest Editor, is given below to highlight the quality and significance of these original research studies.

Coating technologies have been widely used for corrosion protection of automobile bodies made of steel sheets. Steel sheet surfaces are usually treated by phosphating before painting. Advanced high-strength steels (AHSS) that are currently used in automobiles for weight reduction contain a significant amount of Si. A Si oxide film on an AHSS surface can act as a barrier to phosphating. In the work reported in [2], Cho et al. studied the effectiveness of different pickling solutions for removing Si oxide from the surface and improving the phosphatability of AHSS. The results show that the optimal pickling solution was HNO_3_-based solution with a HNO_3_ concentration higher than 13%. The corrosion resistance of phosphate-treated AHSS was better using the HNO_3_-based pickling condition than using the HCl-based pickling condition. This research highlights the importance of pre-treatment in affecting the quality and corrosion performance of the final coating product. In the work reported in [3], Ulbrich et al. studied the tribocorrosion and abrasive wear behaviour of another AHSS steel, i.e., hot-formed 22MnCrB5 steel. The steel was coated with an AlSi coating to prevent oxidation during the hot-forming process. The results show that, compared to the cold-formed state, hot forming followed by appropriate cooling resulted in the formation of a hard and tough martensitic/bainitic structure, which possessed much better corrosion resistance and tribocorrosion resistance in 3.5% NaCl solution.

Surface engineering and coatings are commonly applied to tool steels to improve the performance and service life of cutting and forming tools. In the work reported in [4], Wang et al. prepared Mo coatings on H13 tool steel via the electrospark deposition (ESD) process and studied the mechanical and corrosion properties of the resultant coatings. Under optimal conditions, it was possible to produce a coating of about 35 μm thick without severe cracks. The coating possessed a high hardness of nearly 1400 HV and a good wear resistance that was seven times higher than that of the substrate. In particular, the corrosion resistance of the coating was much better than that of the substrate due to the excellent corrosion resistance of Mo. The combination improvement in hardness, wear resistance and corrosion resistance would be beneficial in enhancing the working performance of H13 steel dies and molds in practice.

In many applications, composite coatings with multiple elements and phases are used. The electrochemical responses of different elements and phases will affect the corrosion properties of composite coatings. In the work reported in [5], Parchovianská et al. investigated the hydrothermal corrosion behavior of double-layer glass/ceramic composite coatings with passive fillers. The coatings were produced using the polymer-derived ceramic (PDC) synthesis route, and this type of PDC-produced glass/ceramic coatings are suitable for the protection of stainless steel from oxidation at temperatures up to 950 °C [6]. It was found that the composite coating containing polycrystalline Al_2_O_3_-Y_2_O_3_-ZrO_2_ (AYZ) as an additional filler performed the best. This indicates the potential of PDC coating with AYZ passive filler for applications in harsh environmental conditions.

Galvanizing, or zinc coating, has been commonly used to provide long-term protection against corrosion of steels. However, one of the weaknesses of zinc coating is its relatively low hardness, which affects its sustainable use. Jedrzejczyk and Szatkowska [7] investigated the influence of heat treatment on the hardness and corrosion resistance of hot-dip zinc coating on steel bolts. The Design of Experiments (DOE) approach was used to design the heat treatment experiments. The heat treatment led to the formation of Fe-Zn intermetallic compounds in the coating and, thus, an increased coating hardness from 52 to 204 HV. The authors of [7] demonstrated that through proper selection of the heat treatment conditions, an increase in coating hardness did not lead to a reduction in corrosion resistance, thus maintaining the protective ability of the coating. In many application situations involving the use of galvanized steels, the joining of steels is required and adhesive bonding has become increasingly used. In the work reported in [8], Li et al. studied the interfacial interaction and corrosion resistance between epoxy adhesive and metallic oxide on galvanized steel through a combination of experiments and molecular dynamics (MD) simulation. Their work [8] provided experimental and theoretical evidence regarding atomic bonds at the joint interface.

Surface engineering and coating technologies are also widely used to improve the tribological behavior of titanium alloys, which may also impact corrosion resistance. In the work reported in [9], Zhang et al. studied the corrosion behavior of nitride layer produced on Ti6Al4V alloy through hollow cathodic plasma source nitriding. They found that the nitride layer comprised several sublayers, including a TiN surface top layer, a Ti_2_N interlayer, and an interstitial solid-solution α-Ti(N) diffusion zone [9]. Detailed electrochemical corrosion measurements were conducted in Hank’s solution. The results showed that the TiN top layer had a better ability for passivation than the untreated alloy, thus leading to a significant reduction in corrosion rate. On the other hand, the Ti_2_N interlayer showed deteriorated corrosion resistance when compared to the top TiN layer. This work provides a useful reference for controlled nitriding to achieved optimized corrosion resistance of nitrided titanium alloy. Another category of techniques that has been applied to titanium alloys is laser surface treatments. In the review paper contributed by Wu et al. [10], the authors discussed the fundamentals of the laser shock processing (LSP) technique and its applications to aeronautical materials for improving their wear and corrosion resistance. LSP has many advantages over other mechanical surface-strengthening techniques and can bring about several changes to material surfaces without altering the chemical composition, including grain refinement in the surface region, generation of compressive residual stress to a greater depth, and enhancement of hardness. This review [10] provides a useful reference for researchers to further explore the LSP technique and its application in engineering.

Polymer electrolyte membrane fuel cells (PEMFCs) are one of the promising candidate technologies in vehicles in the fight against greenhouse gas emissions. The bipolar plate is one of the most important components in PENFCs and is exposed to an aggressive environment and corrosive operating conditions. Surface engineering and coating technologies have been used to protect the surface of bipolar plates. In the work reported in [11], Kim et al. deposited a Nb coating on stainless steel, followed by N^+^ ion implantation. The authors studied the composition, structure and corrosion performance of the duplex-treated material and found that N+ implantation effectively modified the deposited Nb coating to form an improved protective film with much enhanced corrosion resistance for the metal bipolar plate. This work provides new possibilities for surface modification to improve fuel cell performance and for other applications [11].

There are two contributions relating to corrosion inhibitors in this Special Issue. In the review paper by Wang et al. [12], the authors provided a review on the recent progress of polymer corrosion inhibitors, including natural polymer-based inhibitors and synthetic polymeric inhibitors, with more focus on the structure designs and applications of synthetic polymeric and related hybrid/composite inhibitors. In another review paper contributed by Shapagina and Dushik [13], the application of electrophoretic deposition (EPD) to form an inhibited polymer film on metals for corrosion protection is discussed. The authors highlighted the methods of forming protective films and coatings on metal surfaces from aqueous solutions and demonstrated that EPD is a promising method for modifying metal surfaces to fight against corrosion.
